# How to reach haze control targets by air pollutants emission reduction in the Beijing-Tianjin-Hebei region of China?

**DOI:** 10.1371/journal.pone.0173612

**Published:** 2017-03-10

**Authors:** Feng Xu, Nan Xiang, Yoshiro Higano

**Affiliations:** 1 School of Economics and Management, Beijing University of Chemical Technology, Beijing, China; 2 Key Research Base of Cultural Relics Protection Technology and Evaluation, State Administration of Cultural Heritage, Beijing, China; 3 College of Economics and Management, Beijing University of Technology, Beijing, China; 4 Research Base of Beijing Modern Manufacturing Development, College of Economics and Management, Beijing University of Technology, Beijing, China; 5 School of Life and Environmental Sciences, University of Tsukuba, Tsukuba, Japan; Nankai University, CHINA

## Abstract

Currently, Haze is one of the greatest environmental problems with serious impacts on human health in China, especially in capital region (Beijing-Tianjin-Hebei region). To alleviate this problem, the Chinese government introduced a National Air Pollution Control Action Plan (NAPCAP) with air pollutants reduction targets by 2017. However, there is doubt whether these targets can be achieved once the plan is implemented. In this work, the effectiveness of NAPCAP is analyzed by developing models of the statistical relationship between PM_2.5_ concentrations and air pollutant emissions (SO_2_, NO_x_, smoke and dust), while taking into account wind and neighboring transfer impacts. The model can also identify ways of calculating the intended emission levels in the Beijing–Tianjin–Hebei area. The results indicate that haze concentration control targets will not be attained by following the NAPCAP, and that the amount of progress needed to meet the targets is unrealistic. A more appropriate approach to reducing air emissions is proposed, which addresses joint regional efforts.

## Introduction

Haze is one of the most long-standing and important environmental problems in China today. In January 2013, for instance, haze affected most parts of northern and eastern China, encompassing an area of over 1.3 million km^2^ and affecting an estimated population of 850 million people [1]. Haze comprises atmospheric particulate matter (PM); fine particles less than 2.5 micrometers in diameter (PM_2.5_) is considered particularly fatal as it can penetrate deep into the lungs, substantially increasing lung cancer per increasing 10 μg/m^3^ [2]. The capital region, including Beijing, Tianjin, and Hebei provinces in northeastern China, is the most polluted area in China. Among 11 prefecture-level cities in Hebei province, 7 cities were ranked top 10 cities of China with highest annual average PM_2.5_ concentration during 2013 and 2014. Beijing and Tianjin were ranked 13^th^ and 11^th^, respectively, in 2013. Furthermore, the daily average PM_2.5_ concentrations in Shijiazhuang and Xingtai in Hebei Province were 155.2 and 148.5 μg/m^3^ in 2013, respectively, almost four times of the national air quality standard of 35 μg/m^3^ to be introduced in 2016 [3]. The World Health Organization Air quality guideline standard (2013) is 10 μg/m^3^; the U.S. Environmental Protection Agency standard is 12 μg/m^3^; the European Union standard is 25 μg/m^3^; and the standard in India is 40 μg/m^3^ [4]. Atmospheric pollution is a fundamental health issue that also affects sustainable economic development; consequently, it is urgently required by all of society to control haze, especially in the Beijing–Tianjin–Hebei region [5].

Numerous epidemiologic and toxicological studies suggest that exposure to PM_2.5_ leads to adverse health effects, particularly cardiovascular and respiratory diseases, in addition to premature death [6]. There exists a causal relationship between short- and long-term PM_2.5_ exposure, cardiovascular effects, and mortality. A causal relationship is also likely to exist between PM_2.5_ exposure and respiratory effects [7]. However, these research results are derived mainly from studies in developed countries and cannot be easily extrapolated to developing countries [4]. Although many studies of air pollution related health issues have been conducted in China since the 1990s [8], most have focused on PM_10_, SO_2_, and NO_2_, rather than PM_2.5_. This is partially due to the lack of routine monitoring data, since PM_2.5_ was not considered in the Chinese Ambient Air Quality Standards until 2012.

To eliminate haze, the Chinese State Council issued the National Air Pollution Control Action Plan (NAPCAP) in September 2013. According to the NAPCAP, Chinese government will strive to improve the country’s seriously polluted atmosphere and national air quality over the next five years and beyond. This involves specific targets, including reductions of approximately 25%, 20%, and 15% in PM_2.5_ in the Beijing–Tianjin–Hebei, Yangtze Delta, and Pearl River Delta areas, respectively, and the annual average PM_2.5_ in Beijing being below 60 μg/m^3^ by 2017[9]. NAPCAP defines 10 measures to achieve these objectives. Additionally, the Beijing–Tianjin–Hebei region has proposed specific local action plans to reduce atmospheric emissions. These measures focus on the overall treatment of multiple pollutants, adjusting and optimizing energy and industrial structure, and promoting economic structure transition.

Haze is an atmospheric phenomenon whereby dust, smoke, and other dry particles obscure the clarity of the sky. Haze is usually created by physical and chemical processes, involving fine particulate matter interacting with water vapor under certain airflow conditions. Haze occurs when PM_2.5_ levels in the atmosphere are high. As serious haze events have occurred more frequently in China in recent years, regional PM_2.5_ limits have now been included in the Ministry of Environmental Protection’s 2012 Ambient Air Quality Standard (GB3095-2012) as a national ambient air quality standards index.

**Fig 1:**
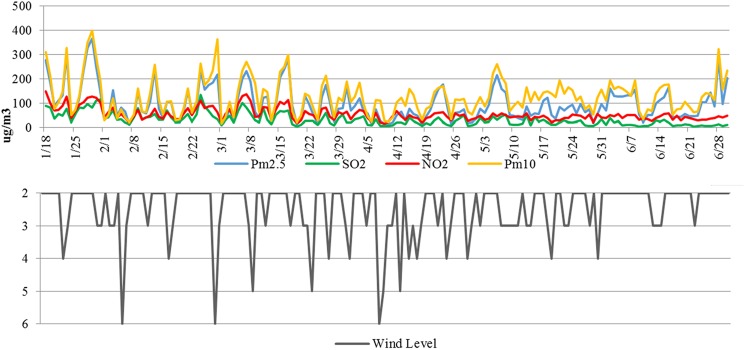
2013 daily average PM_2.5_, air pollutant concentration and wind levels in Beijing.

In recent years, there has been an increasing amount of research related to haze and PM_2.5_ reduction and control, including chemical and compositional analysis, environmental control strategy analysis, and health risk analysis (Table 1). A fundamental research area is the study of the composition of haze and decomposition of the factors affecting haze, which mainly focuses on the physical and chemical characteristics of PM_2.5_ to understand the essence of haze. Chemical analysis of PM_2.5_ is usually used to identify emission sources. The U.S. Air Forces Southern Command has proposed that the total amount of PM_2.5_ is the sum of precursor pollutant emissions and secondary particles; the precursor pollutants include sulfur dioxide (SO_2_), nitrogen oxides (NO_x_) and direct PM_2.5_; and secondary particles comprising various compounds generated by precursor pollutants [10]. PM_2.5_ control can be solved by reducing emission of SO_2_, NO_x_, volatile organic compounds, and ammonia. Due to the limited statistical data in China, PM_2.5_ composition analysis typically identifies PM_2.5_ as a compound pollutant related to SO_2_, NO_x_, smoke, and dust emissions [11]. Fig 1 shows the factors involved in PM_2.5_ decomposition.

**Table 1:** Types of research related to haze.

Types	Category
**PM2.5 composition research**	1. Chemical analysis
	2. Social and economic source apportionment
**Environmental control strategies on PM2.5 and haze**	**1. Air quality model**: Simulating the effect of chemical elements and atmospheric conditions on PM2.5 concentration (such as CMAQ model);Assessing atmospheric environmental capacity
	**2. Statistical model**: The relationship of PM2.5 and air pollutant concentration; Scenario analysis based on statistical model estimation
**Health effects**	1. Epidemiological and toxicological studies of developed countries 2. Influence of PM2.5 on human health in Chinese cities

Researchers have also used quantitative analysis of pollutant emissions to identify energy consumption and vehicle combustion engines as major pollution sources. Previous studies have analyzed PM_2.5_ decomposition using multiple linear regression, principal component analysis, and mass balance analysis in decomposing PM_2.5_ into chemical elements to identify the main sources of haze, in order to control air pollutant emissions [12–15].

Haze and air pollutants are easily transported between neighboring areas. For example, vehicle and oil consumption factors have been shown to account for 25% of PM_2.5_ sources in Beijing; transport from neighboring regions contributing up to 19% [1]. Although these high contributions due to neighboring transport suggest the need for regional joint PM_2.5_ prevention and control, to our knowledge there have been rare studies on haze control from a regional perspective.

Analysis of PM_2.5_ composition for prevention and control strategies of haze involves two methods, which are distinct by modeling approaches—air quality modeling or statistical modeling. Air quality models are usually used to simulate chemical elements and their effects on atmospheric conditions like PM_2.5_ concentration [16–17]. Multivariate statistical methods are used to simulate and forecast PM_2.5_ control by depicting the relationship between air pollutants and PM_2.5_. For such studies, air pollutant emission sources are usually identified by construction of a statistical model based on the chemical proportions involved [16–19].

It is critical to control PM_2.5_ emissions in China, as haze can cause significant damage to human health. Prior to the haze event in January 2013, the Chinese government utilized reduction targets for air pollutant emissions to control air quality, without regulating PM_2.5_ concentrations. In 2013, the Chinese government changed their air quality control indicator from total emissions amount management to concentrations control, leading to concentration [μg/m^3^] targets being proposed in NAPCAP. However, there is limited research related to the relationship between PM_2.5_ concentration and its precursors, SO_2_, NO_x_, and smoke and dust, making it difficult to evaluate whether the current concentration targets are attainable through air pollutant reduction plans.

In short, haze prevention research should focus on reducing the concentration of PM_2.5_ by air pollutant emission control. Given the limited availability of data, this can be achieved by controlling PM_2.5_ precursors, SO_2_, NO_x_, smoke, and dust. However, the link between PM_2.5_ concentrations and the concentrations of these precursors has yet to be clearly established, making it difficult to predict and evaluate their effect on haze control and to develop practical implementation goals and strategies. This study aims to analyze the statistical relationship between haze concentration and air pollutant emissions based on data from the Beijing–Tianjin–Hebei region since 2013, in order to convert the target of a 25% reduction in PM_2.5_ (for Beijing, it is maximum 60 μg/m^3^) into air pollutants emission reduction targets. This will make it possible to utilize the relationships involved to estimate whether the current air pollutants emission reductions plan can meet PM_2.5_ targets.

This paper contributes to methodological improvements and practical evaluation of NAPCAP. Descriptive analyses is used to determine the major factors affecting PM_2.5_ concentrations in the Beijing–Tianjin–Hebei region, including air pollutant emissions, wind, and neighboring transfer impacts. A statistical model is then constructed for each region to demonstrate the regional haze formation and its impacting factors, and to examine the relationship between PM_2.5_ concentrations and air pollution emissions. Specially, factor analysis is used to solve the collinearity problem of major air pollutant daily emissions, and a quantile correction model is utilized to forecast data for extreme value correction. The resulting verified and fitted models can effectively predict PM_2.5_ concentrations that result from different strategic air emission reduction scenarios under a variety of conditions, thereby enabling us to evaluate and estimate the likely success of NAPCAP in the Beijing–Tianjin–Hebei region.

## Instruments and measurements

### Descriptive data analysis

The currently available descriptive data is analyzed in order to identify the major factors affecting PM_2.5_ concentrations in each region. Data on air pollutants emissions are collected from the regional Statistical Yearbook and China Energy Statistical Yearbook, which include the annual SO_2_, NOx, and smoke and dust emissions for the 13 cities in the Beijing–Tianjin–Hebei region. The daily concentration level of PM_2.5_ and air pollutants, is extracted from environmental monitoring data (13 cities, 80 environmental monitoring points) provided by Greenpeace-East Asia (Beijing Office). Since Beijing’s urban and suburban area have significant difference on air pollutants emissions sources and vehicle amount, Beijing city is divided into urban and suburban observation regions. Therefore, air pollutants emission and PM_2.5_ concentrations from 14 regions are studied in this work.

According to Chinese national standards, the daily average PM_2.5_ concentration limit over one year is 35 μg/m^3^(Level 1 standard for air quality), and 75μg/m^3^(Level 2 standard) over an individual 24-hour period for acceptable air quality. As Table 2 shows, the daily average concentration over one year in the study region is exceeded by up to four times the limit and over 50% of the days are higher than the 24-hour maximum. The haze problem in this region, therefore, ranges from significant to extreme, with Shijiazhuang, Xingtai, Handan, and Baoding being highly polluted (Table 3).

**Table 2:** Daily average PM_2.5_ concentration of Beijing- Tianjin-Hebei Region in 2013.

Range	PM2.5 concentration	Ratio	Cumulative ratio
**<35μg/m^3^**	20.64	19.32%	19.32%
**35–75μg/m^3^**	54.38	29.89%	49.21%
**75–150μg/m^3^**	105.86	34.18%	83.39%
**150–200μg/m^3^**	172.48	8.08%	91.47%
**>200μg/m^3^**	280.63	8.53%	100.00%

Source: Greenpeace-East Asia (Beijing Office)

**Table 3:** Daily average PM_2.5_ and air pollutants concentration of Beijing- Tianjin-Hebei Region.

Region	Annual daily average concentration(μg/m^3^)				PM_2.5_/PM_10_
	PM_2.5_	SO_2_	NO_2_	PM_10_	
**Urban Beijing**	89.70	33.61	48.00	113.99	78.69%
**Suburban Beijing**	75.80	19.17	35.02	97.68	77.60%
**Tianjin**	92.34	48.86	47.26	154.03	59.95%
**Baoding**	120.19	55.55	50.33	212.43	56.58%
**Langfang**	101.70	37.37	43.72	171.45	59.32%
**Zhangjiakou**	40.30	41.02	27.17	94.79	42.52%
**Tangshan**	110.42	92.65	63.10	181.41	60.87%
**Chengde**	50.93	31.22	31.78	99.84	51.01%
**Shijiazhuang**	142.43	93.73	60.91	287.63	49.52%
**Qinhuangdao**	63.80	53.87	45.50	125.47	50.85%
**Cangzhou**	92.12	49.29	31.22	127.63	72.18%
**Hengshui**	112.68	56.55	40.88	203.13	55.47%
**Xingtai**	140.13	67.48	47.20	209.39	66.92%
**Handan**	126.59	84.85	55.49	226.13	55.98%
**Average**	96.94	54.66	44.83	164.64	58.88%

Source: Greenpeace-East Asia (Beijing Office)

A strong correlation exists between the concentration of pollutants and wind speed (Fig 1). A large regional disparity is observed in the Beijing–Tianjin–Hebei area, with haze being heavier in the south than the north (Table 4). Cities in the northern region, such as Zhangjiakou, Chengde, and Qinhuangdao, have better air quality, with annual average PM_2.5_ concentrations of <65 μg/m^3^ in 2013. This is attributed to lower pollutant emissions and larger air flow capacity in these regions. Furthermore, cities with larger air pollutants emission amount in southern part of the Beijing–Tianjin–Hebei area has much higher PM2.5 concentration, such as Shijiazhuang, Hengshui, Xingtai, and Baoding.

**Table 4:** 2013 PM_2.5_ concentration with different wind levels.

Region	PM_2.5_ annual concentration (μg/m^3^)				
	Less than level 2	Level 3	Level 4	Level 5	Larger than level 5
**Urban Beijing**	103.86	**58.65**	**33.19**	**21.33**	**10.25**
**Suburban Beijing**	87.47	**52.04**	**25.41**	**18.04**	**8.94**
**Tianjin**	112.95	92.82	**65.36**	**30.67**	**17.21**
**Baoding**	129.5	87.77	**53.8**	**41.46**	**-**
**Langfang**	115.19	**68.33**	**46.05**	**25.34**	**63.15**
**Zhangjiakou**	**49.54**	**44.29**	**39.04**	**30.2**	**22.72**
**Tangshan**	167.52	115.02	78.34	**53.52**	**-**
**Chengde**	**56.65**	**41.98**	**38.77**	**22.96**	**-**
**Shijiazhuang**	183.42	113.02	77.14	**38.44**	**24.89**
**Qinhuangdao**	83.41	**68.87**	**54.68**	**23.34**	**17.69**
**Cangzhou**	111.46	96.09	84.77	**48.97**	**32.33**
**Hengshui**	120.03	85.58	**68.71**	**54.25**	**-**
**Xingtai**	121.12	89.46	**67.41**	**54.21**	**-**
**Handan**	137.79	120.06	92.76	**42.58**	**30.75**

Note: Bold figures in this table demonstrate the acceptable PM2.5 concentrations(less than 75μg/m^3^).

**Fig 2:**
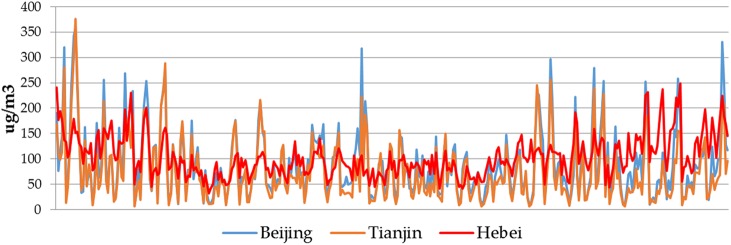
2013 regional PM_2.5_ concentration trends.

PM_2.5_ trends in the Beijing–Tianjin–Hebei region are closely linked among the various sub-regions, as higher concentrations are often observed at the same time (Fig 2). As discussed above, 19% of pollutants in the heavy haze event of January 2013 were originated from outside the region, thereby highlighting the importance of analyzing neighborhood effects in this study.

Briefly, the descriptive data analysis indicates that air pollutants and neighborhood effects have a positive impact on PM_2.5_ concentrations, whereas wind level has a significantly negative effect. In addition, air pollutant emissions are the fundamental reason for the occurrence of haze.

### Econometric specification and method

Considering the main factors affecting PM_2.5_ concentrations, this study constructs regression models to characterize the relationship with air pollutants emissions, wind, and regional effects [20] using the following equation:

Y=f(X,lY,lNY,lSY,LowlW,MediumlW) (1)

where Y denotes the PM_2.5_ concentration in each region (PM_2.5_ daily average data are summarized from environmental monitoring data of 80 monitoring sites of the Beijing-Tianjin-Hebei region of China, which are provided by Greenpeace-East Asia (Beijing Office)).

*X* is the principal components index for the daily emission density of SO_2_, NO_x_, and smoke and dust emissions in each region. Given the strong correlation between the three air pollutant emissions, factor analysis is used to extract the principal components into the model as *X* to solve multi-collinearity problems. Also, to facilitate comparisons between regions, emission density (i.e., the emissions per unit area) is used to demonstrate the effect of air pollutant emissions in *X*.

*lY* is the lag concentration of PM_2.5_ characterizing the cumulative effect of pollutants in the air.

*lNY* and *lSY* are the lagged PM_2.5_ concentrations adjacent to the northern and southern areas, respectively, characterizing the role of air pollutant transportation between neighboring regions. When the wind is from the northeast/north/northwest, the northern adjacent region’s influence on concentration PM_2.5_ is calculated; otherwise, it is zero.

Wind is divided into three grades: ‘low’ for a low-speed wind and light breeze (level2), ‘medium’ for third and fourth wind level, and ‘high’ for wind stronger than level five. Wind levels are represented by two dummy variables: *LowlW* is the lagged low-speed wind and *MediumlW* is the lagged medium-speed wind; high-speed wind is set as the reference group in the model. Wind speed and direction are also set for neighboring regions. Given the strong positive correlation between wind and rainfall in this region, and low rainfall frequency, the statistical regression model does not consider air temperature or rainfall (which are usually treated here as dummy variables).(Wind data is collected from China Weather Net, http://www.weather.com.cn/)

For the value of *X* in each region, the daily air pollutants emission concentration is estimated based on the annual emissions of the various pollutants, reference heating data(open accessed and collected from China Energy Statistical Yearbook), fluctuations in electricity consumption, and indicators of traffic congestion. The 14 regional annual air pollutants emission concentrations are subdivided into daily emission concentrations using the following method.

Step 1: Distinguish air pollutant emissions of heating and non-heating seasons. Obtain the added heating-season air pollutant emissions based on each region’s consumption of heating energy.

Step 2: Calculate monthly air pollutant emissions in each area using the industrial production prosperity index to reflect the monthly fluctuations in socio-economic activities (National Bureau of Statistics of China).

Step 3: Estimate daily emissions data based on the different production capacities on weekdays, weekends, and holidays. Socio-economic activities are different for each day, and the total electricity consumption indicator can demonstrate disparity in economic activity among the days. Guotai Junan reference experience indicators (Guotai Junan Securities “Report on Electricity Industry”) are used for this purpose, setting weekend days approximately equal to 0.92 work days and holidays approximately equal to 0.75 work days. In this way, monthly emissions are downscaled to daily emissions.

Step 4: Correct the daily air pollutants emission data for Beijing, since Beijing has more than 500 million vehicles and heavier traffic jam. Data correction uses the Beijing congestion factor (data from Beijing Municipal Commission of Transport). Vehicle emissions account for a relatively high ratio of Beijing air pollutants due to urban traffic congestion, which creates higher emissions due to inefficient fuel combustion. Extracted data from 18/01/2013-30/06/2013 of 14 regions in this study can be accessed in S1 Table.

### Estimation results

Table 5 lists the results of the time series regression model for each region from January 2013 to June 2014. Missing data are not included in the analysis. Overall, the various factors have significant impacts on PM_2.5_ concentrations and differ for each region.

**Table 5:** Model estimate results.

Variables	Urban Beijing		Tianjin		Baoding		Tangshan		Shijiazhuang	
*X*	0.019	*	0.049	***	0.277	***	0.036	***	0.221	***
*lY*	0.444	***	0.419	***	0.526	***	0.334	***	0.344	***
*lNY*	0.011		0.055		0.182	**	0.187	***	0.229	***
*lSY*	0.146	**	0.148	***	0.095	*	0.321	***	0.39	***
*LowlW*	73.586	***	69.846	***	89.204	***	93.465	***	72.91	***
*MediumlW*	32.3	**	41.368	***	33.616		61.462	***	31.334	
*Constant*	-40.035	**	-29.376	***	-75.458	**	-39.969	***	-46.698	
**R2**	0.42		0.466		0.521		0.385		0.48	
**DW value**	1.79		1.965		1.799		1.874		1.914	
**Sample number**	501		495		469		500		495	
Variables	Cangzhou		Hengshui		Xingtai		Handan		Qinhuangdao	
*X*	0.293	***	0.14	***	0.134	*	0.236	***	0.059	**
*lY*	0.369	***	0.277	***	0.637	***	0.564	***	0.384	***
*lNY*	0.366	***	0.553	***	0.154	***	0.068	*	0.119	*
*lSY*	0.5	***	0.482	***	0.072		0.158	***	0.068	*
*LowlW*	40.699	***	22.988	**	41.692	*	45.321	**	45.759	***
*MediumlW*	24.7	***	9.785		12.863		21.752		23.644	***
Constant	-19.229	*	-29.805	**	-21.691		-39.679	*	-10.44	
**R2**	0.355		0.71		0.561		0.569		0.349	
**DW value**	1.827		1.942		1.849		1.864		1.901	
**Sample number**	494		495		433		495		495	
Variables	Beijing surburbs		Langfang		Zhangjiakou		Chengde			
*X*	0.019	*	0.242	***	0.135	**	0.569	***		
*lY*	0.449	***	0.502	***	0.629	***	0.553	***		
*lSY*	0.141	***					0.041			
*LowlW*	73.561	***	80.979	***	21.014	***	29.681	***		
*MediumlW*	32.273	**	34.375	**	10.446	***	14.369	**		
*Constant*	-39.968	**	-59.916	***	-8.198		-25.349	***		
**R2**	0.454		0.429		0.506		0.444			
**DW value**	1.775		1.856		1.727		1.788			
**Sample number**	499		503		502		500			

*t* statistics with “* p<0.10 ** p<0.05 *** p<0.01”

The variables’ DW values demonstrate there is no autocorrelation.

Local air pollutants emissions have a significant impact on PM_2.5_ concentrations, as well as local lagged PM_2.5_ concentrations, reflecting the cumulative effect of pollutants in the atmosphere. Compared with high-speed wind, low-speed wind has an obvious cumulative effect on air pollutants, hence increasing PM_2.5_ concentrations, especially in areas with large air pollutant emissions. In general, PM_2.5_ concentrations in neighboring areas have a significant effect on regional air pollutants flow, thereby confirming the role of interregional transport. Given the variables of each region’s geographical and weather conditions, the regional effects of air pollutant transportation differ between each region, with suburban Beijing and Chengde being strongly affected by adjacent areas to the south, whereas Zhangjiakou and Langfang being weakly affected.

Based on the results and the coefficients listed in Table 5, accuracy of each model is assessed by comparing the predicted and measured values. Results indicate the models to be generally reliable for suburban Beijing, Tianjin, Baoding, Langfang, Tangshan, Qinhuangdao, Cangzhou, and Handan. The estimates for the other regions are less accurate since they fail to predict extreme values. This problem can be corrected by quantile regression, by including the highest 10% value (90% quantile) and lowest 10% value (10% quantile) of the PM_2.5_ concentrations (Table 6).

**Table 6:** Regional quantile regression results (90% and 10% quantile).

	Variables	**Urban Beijing**		**Tianjin**		**Baoding**		**Langfang**	
90% quantile	*X*	0.044	**	0.048		0.457	***	0.425	***
	*lY*	0.303		0.297	***	-0.077				0.836	***
	*lNY*	0.355		0.137	*	0.242	*		
	*lSY*	0.658	**	0.572	***	0.919	***		
	*LowlW*	89.784	***	78.141	***	69.693		87.001	***
	*MediumlW*	34.179	***	40.952	***	4.736		37.294	***
	*Constant*	-51.603	**	-10.121		-47.668		-68.494	***
	Variables	**Tangshan**		**Qinhuangdao**		**Cangzhou**			
	*X*	0.063	**	0.097		0.293	***		
	*lY*	0.588	***	0.152		0.369	***		
	*lNY*	0.633	***	0.167		0.366	***		
	*lSY*	0.483	***	0.660	***	0.500	***		
	*LowlW*	145.903	*	57.342	***	40.699	***		
	*MediumlW*	69.908	***	31.064	**	24.700	***		
	*Constant*	-58.216	**	-9.592		-19.229	*		
	Variables	**Urban Beijing**		**Baoding**		**Shijiazhuang**			
10% quantile	*X*	0.0078		0.1022		0.0392			
	*lY*	0.1169		0.1539		0.1381			
	*lNY*	0.0233		0.21786		0.1303			
	*lSY*	0.0869	*	0.0962		0.1886			
	*LowlW*	40.25	**	67.275	**	18.5195	***		
	*MediumlW*	19.44		43.237		2.308			
	*Constant*	-23.92		-50.624	*	1.6347			

*t* statistics with“* p<0.10 ** p<0.05 *** p<0.01”

**Fig 3:**
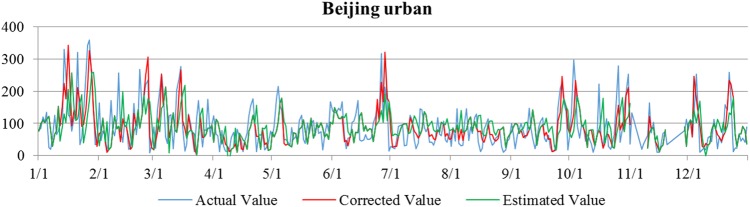
Comparison of actual, predicted, and corrected values in the urban Beijing area

**Fig 4:**
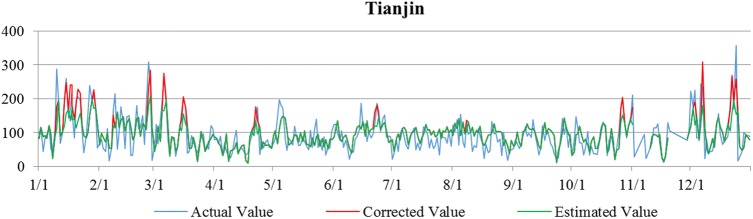
Comparisons of actual, predicted, and corrected values in the Tianjin area

Based on the deviation changes between the actual, the estimated, and corrected values (Figs 3 and 4), it is concluded that quantile regression significantly improves the accuracy of these models, allowing them to be used in assessing the feasibility of NAPCAP to achieving PM2.5 concentration targets.

## Evaluation of the NAPCAP

### Measurement evaluation

This study uses daily air pollutants emissions to calculate daily pollutant concentrations, while annual air pollutants reduction plan is adopted from Clean Air Alliance of China’s calculation based on NAPCAP compared with current emissions [3]. Daily air pollutants emissions data is calculated based annual amount; then the model constructed in this study estimates daily PM_2.5_ concentrations, taking into account control variables such as wind and neighborhood effects.

Table 7 compares the PM_2.5_ concentrations predictions can be achieved by NAPCAP and the set reduction targets for 14 regions of the Beijing–Tianjin–Hebei region. Simulation results show that the reduction targets will not be attained without reducing air pollutants in surrounding regions. In addition, even if the surrounding areas are able to reduce their air pollutant emissions, the PM_2.5_ concentrations targets will only be achievable in Chengde, Shijiazhuang, and Handan.

**Table 7:** PM_2.5_ concentration estimation based on NAPCAP (Unit: μg/m^3^).

Region	NAPCAP air pollutants reduction plans	Model prediction of concentration level at current rate of progress: with *reduced* air pollutants in surrounding area		Model prediction of concentration level at current rate of progress: with *unreduced* air pollutants in surrounding area	
		PM_2.5_ concentration	Reduction achieved	PM_2.5_ concentration	Reduction achieved
Urban Beijing	63%	66.35	26%	71.51	21%
Suburban Beijing	63%	49.57	35%	53.39	30%
Tianjin	37%	82.77	10%	85.79	7%
Baoding	29%	100.04	17%	103.31	14%
Langfang	29%	92.20	9%	-	-
Zhangjiakou	29%	30.53	24%	-	-
Tangshan	29%	94.83	14%	104.35	6%
Chengde	29%	35.53	30%	37.15	27%
Shijiazhuang	29%	96.24	33%	120.28	16%
Qinhuangdao	29%	54.96	14%	59.16	7%
Cangzhou	29%	75.17	18%	90.12	1%
Hengshui	29%	92.67	18%	108.76	1%
Xingtai	29%	111.11	21%	127.28	9%
Handan	29%	90.20	29%	103.52	18%

Based on the predicted absolute concentration levels, only Chengde and Zhangjiakou are on track to comply with the national PM_2.5_ standard of 35μg/m^3^. With the help of emissions reductions in surrounding areas Urban Beijing is expected to achieving its 60μg/m^3^ target. Many other regions, including, Baoding, Langfang, and Tangshan, are predicted to have PM_2.5_ levels that are three times of the national standard.

These results indicate that at the current progress, the NAPCAP targets will be very difficult to achieve, if not impossible. Tianjin and Hebei need to further intensify their efforts to reduce emissions, while Shijiazhuang, Baoding, Tangshan, Handan, Xingtai, Hengshui, and other areas will not meet the national standards by solely decreasing the average annual PM_2.5_ concentrations by 25% as recommended by the NAPCAP.

### Permitted air pollutants emissions according to PM_2.5_ control targets

According to the targets for PM_2.5_ concentration reductions set by the NAPCAP, Tianjin and Hebei need to decrease concentrations by 25% compared with 2012. Unlike other cities, Beijing has a higher standard set at 60 μg/m^3^. This study utilizes the model to predict permitted air pollutants emission amount which can satisfy PM_2.5_ concentration reductions targets; Table 8 lists the predicted PM_2.5_ concentration targets (column 2) needed by applying the NAPCAP 25% target (column 3) with an assumed split of 30% and 20% for urban and suburban Beijing, respectively. Column 4 in Table 8 provides the percentage reduction in air pollutants (SO_2_, NO_x_, and smoke and dust) needed to meet the required concentration levels.

**Table 8:** Air pollutant reductions requirement to realize the NAPCAP PM2.5 targets (Unit: μg/m^3^).

Region	Reduced air pollutant in surrounding area		Air pollutants reduction needed to meet NAPCAP μg/m^3^ targets	Unreduced air pollutant in surrounding area	
	PM_2.5_ concentration	NAPCAP PM_2.5_ reduction target		PM_2.5_ concentration	Reduce rate
Urban Beijing	60.29	assumed 30%	**78%**	64.94	28%
Suburban Beijing	60.33	assumed 20%	**45%**	65.39	14%
Tianjin	69.66	25%	**61%**	72.58	21%
Baoding	90.24	25%	**40%**	93.21	22%
Langfang	76.48	25%	**44%**	-	-
Tangshan	82.88	25%	**52%**	92.15	17%
Shijiazhuang	106.83	25%	**15%**	128.22	10%
Cangzhou	68.81	25%	**52%**	86.69	6%
Hengshui	84.76	25%	**48%**	110.10	2%
Xingtai	105.03	25%	**37%**	121.20	14%
Handan	92.44	25%	**27%**	81.98	11%

Note: Zhangjiakou, Chengde and Qinhuangdao have relatively good quality of air, with PM_2.5_ concentration less than 65μg/m^3^ in 2013, and this can further be improved with neighboring reduction. Therefore, this model assumed that Zhangjiakou, Chengde and Qinhuangdao do not have to reduce their air pollutants emission.

Table 9 converts these air pollutants reduction rate into permitted emission tons, showing that the Beijing–Tianjin–Hebei region needs to reduce its air pollutant emissions by approximately 42% by 2017. However, despite reductions in PM_2.5_ concentrations, the average annual concentration in some regions (e.g., Shijiazhuang, 106.93 μg/m^3^) remains much higher than the national air quality standard for public health. For long-term planning, reductions in air pollutant emissions need to be further intensified to achieve acceptable air quality standards.

**Table 9:** Allowed air pollutants emission amounts according to PM2.5 reduction targets.

Region	SO_2_ (ton)	NO_x_ (ton)	Smoke and Dust (ton)	Reduction rate
Urban Beijing	9,514	17,211	6,338	78%
Suburban Beijing	24,018	43,448	16,000	45%
Tianjin	81,685	119,070	31,859	61%
Baoding	63,547	96,675	61,872	40%
Langfang	17,703	31,580	20,894	44%
Tangshan	190,118	247,489	183,783	52%
Shijiazhuang	147,291	191,741	93,010	15%
Cangzhou	35,124	45,962	34,043	52%
Hengshui	29,694	37,704	38,066	48%
Xingtai	54,415	62,935	58,934	37%
Handan	21,487	70,572	83,212	27%
**Total**	898,988	1,189,079	779,065	
**Average reduction rate**	41%	43%	41%	42%

## Discussion and conclusions

In 2013, the Chinese government changed its air quality control management strategy, transferred focus on air pollutant emissions control targets to concentration reduction targets (especially for PM_2.5_). However, the relationship between PM_2.5_ concentration and air pollutants emission is unclear, and the effectiveness and feasibility of current reduction measurements cannot be verified without understanding the relationship between air pollutants emissions and PM_2.5_ concentrations. Taking into account these situations, this work developed regional models of the statistical relationship between PM_2.5_ concentrations and air pollutant emissions (SO_2_, NO_x_, Smoke and Dust), as well as taking account with wind and neighborhood transfer effects, in order to evaluate the effectiveness of the current air pollutants reduction plan in attaining the PM_2.5_ targets.

With verified model and estimation, simulations results point out that even though the Beijing–Tianjin–Hebei region take great measures to eliminate haze under the current reduction targets set by NAPCAP, the PM2.5 control targets cannot be easily accomplished. Models indicate the existence of two major problems: first, the targets are not set high enough to bring about the reductions in concentration needed; second, the targets required to meet the concentration reductions are unrealistically high, thereby raising questions about the region’s ability to achieve the targets. For instance, Beijing’s emission reduction targets are particularly difficult to attain, requiring an increase in the rate of pollutant emission reduction from the planned 63% to 78%. Clearly, the PM_2.5_ targets for the Beijing area need to be adjusted to be more realistic and feasible. In some regions in Hebei, the annual concentration target of PM_2.5_ will be still higher than 75 μg/m^3^, which is far beyond human health standards. Due to the social, health, and economic impacts of haze, it is critical for the Chinese government to set reasonable reduction targets as the first step to solve the problem of haze.

Pollutant reduction actions in China are related to regional economic development and livelihood security, making the implementation of haze pollution control strategies a long process before the problem can be solved. As an example, the U.S. first included PM_10_ in its ambient air quality standards in 1987 and then spent nearly 10 years trying to solve the haze problem. Based on the experiences of developed countries, long-term regional efforts are needed to control haze. It is important, therefore, to set realistic PM_2.5_ control targets and to establish comprehensive support plans.

However, the present results indicate that haze prevention and control can be achieved by changing the focus from PM_2.5_ concentration targets to air pollutant emissions management. By using scientific methods to determine the environmental capacity for reduction targets, it will be possible to develop realistic plans for the future. Further studies are needed to propose a reasonable PM_2.5_ improvement plan and air pollutants reduction targets, with consideration of economic cost–benefit analysis. One approach is the introduction of air pollutant permits to help maximize capacity reduction over a specific time period and to minimize health risks, while taking into account the costs and benefits of haze control action plans.

Further research are needed to develop proposals for a sustainable, a concrete, and a reasonable air pollutants emission reduction action plan for haze prevention and control in China. This should include a joint regional air pollutants reduction plan, suitable PM_2.5_ reduction targets and effective social–economic action plans. Once formulated, such a plan would provide a useful example for other developing countries in similar situations with China.

## Supporting information

S1 Table PM2.5 and air pollutants emission data of 14 regions in Beijing-Tianjin-Hebei region (18/01/2013-30/06/2013). (XLSX)
